# *Bartonella* spp. and *Yersinia pestis* Reservoirs, Cusco, Peru

**DOI:** 10.3201/eid2006.131194

**Published:** 2014-06

**Authors:** Aarón Martin-Alonso, Mayday Soto, Pilar Foronda, Elsa Aguilar, Guillermo Bonnet, Rosa Pacheco, Basilio Valladares, Maria A. Quispe-Ricalde

**Affiliations:** University of La Laguna, Canary Islands, Spain (A. Martin-Alonso, P. Foronda, G. Bonnet, B. Valladares, M. A. Quispe-Ricalde);; National University of San Antonio Abad, Cusco, Peru (M. Soto, E. Aguilar, R. Pacheco)

**Keywords:** plague, Bartonella spp., bartonellosis, Yersinia pestis, bacteria, reservoirs, rodents, emerging infectious diseases, zoonoses, rodents, Cusco, Peru

**To the Editor:** *Bartonella* spp. are gram-negative alphaproteobacteria that are transmitted between the reservoir and mammal host by hematophagous insects (*1*). The genus *Yersinia* comprises 11 species, of which *Y. pestis* is the causative agent of plague, a deadly rodent-associated, fleaborne zoonosis (*2*). Despite the large number of plague cases reported in humans and the large amount of data about human-infecting *Bartonella* spp. in Peru (*3*), no data have been published about which rodent species are reservoirs of these pathogens in this country.

La Convención Province, where cases of bartonellosis occurred during 1998 (*4*), is located in the northeastern part of Cusco, Peru. Although, to our knowledge, no human cases of plague have been reported in this province, a plague outbreak was recently detected in Junin Province (M.A. Quispe-Riclade, pers. comm.), which is located northwest of La Convención Province.

A total of 28 rodents were captured during 2010–2011 in 3 villages (Alto Ivochote, Aguas Calientes, and Yomentoni) in the Echarate District, La Convención Province. Traps had been set in intradomiciliary, peridomiciliary, and extradomiciliary settings. Spleens of animals were obtained, and DNA was isolated by using the Illustra Tissue and Cells Genomic Prep Mini Spin Kit (GE Healthcare, Little Chalfont, UK).

Rodents were examined for *Bartonella* spp. DNA by using a PCR and primers CS443f and CS1210r specific for a 767-bp fragment of the citrate synthase gene (*5*). Screening for plague was performed by using PCR primers Yp1 and Yp2 specific for a plasminogen activator protein (*pla*) encoded by the *Y. pestis*–specific pPLA plasmid (*6*).

New and published *Bartonella* spp. and *Y. pestis* sequences were obtained from GenBank and compared by using the nucleotide-nucleotide basic local sequence alignment tool (BLAST) (blastn) program (www.ncbi.nlm.nih.gov/Class/MLACourse/Modules/BLAST/nucleotide_blast.html). The χ^2^ test was used to determine statistical differences in the prevalence of both pathogens among host species and villages.

Overall prevalences for *Y. pestis* and *Bartonella* spp. were 17.9% and 21.4%, respectively (Table). Co-infections with both bacteria were found in 3 (10.7%) rodents: 2 *Hylaeamys perenensis* rodents and 1 *Oecomys* spp. rodent. *Bartonella* prevalence was higher in *H. perenensis* rats than in *Rattus rattus* rodents (p<0.001). Rodents positive for *Bartonella* spp. were found in the 3 study villages, and prevalence for Aguas Calientes was higher than that for Alto Ivochote (p<0.001). One of 8 rodents trapped inside houses and 1 of 2 rodents trapped at peridomestic sites were positive for *Bartonella* spp.

**Table:** Prevalence of *Bartonella* spp. and *Yersinia pestis* in rodents from Echarate District, Cusco, Peru

Study area	Rodent host species (no.)	No. (%) positive for *Yersinia pestis*	No. (%) positive for *Bartonella* spp.
Alto Ivochote	*Rattus rattus* (20)	3 (15)	0 (0)
Alto Ivochote	*Hylaeamys perenensis* (1)	0	1 (100)
Aguas Calientes	*Hylaeamys perenensis* (2)	2 (100)	2 (100)
Aguas Calientes	*Oecomys* spp. (1)	1 (100)	1 (100)
Yometoni	*Rattus rattus* (4)	0	1 (25)
Total	(28)	6 (21.4)	5 (17.9)

Sequence analysis identified 3 citrate synthase gene sequences (GenBank accession nos. KF021602–KF021604) that had 98% and 99% sequence similarity to genotypic variant A3 of the undescribed *Bartonella* genogroup A, which was obtained from *Oryzomis palustris* rats in the southeastern United States (*7*). One genotype (isolate B259) was identified in *H. perenensis* rats, and 2 other genotypes were identified in 1 *H. perenensis* rodent and 1 *Oecomys* spp. rodent (isolates B273 and B280, respectively).We propose that the genotype of isolates B273 and B280 is variant A6 and the genotype of isolate B259 is variant A7. A previous study reported that the A, B, and C genogroups contain independent species (*8*).

The *pla* amplicons (GenBank accession nos. KF214264–KF214266) had 98% sequence identity with *Y. pestis* reference sequences. Plague prevalence was higher in *H. perenensis* rats than in *R. rattus* rats (p<0.05). Infected rodents were found in all villages studied except Yomentoni, and prevalence in Aguas Calientes was higher than in Alto Ivochote (p<0.01). Two (25%) of 8 rodents trapped inside houses were infected with *Y. pestis*.

This study suggests that infections of rodents with *Bartonella* spp. and *Y. pestis* are common and widespread throughout the Echarate District. It also shows the role of *H. perenensis* and *Oecomys* spp. rodents as reservoirs of both pathogens. This role was confirmed by amplifying the chromosomal ferric iron uptake regulation gene by using PCR primers Ypfur1 and Ypfur2, as described by Hinnebusch et al. (*9*). The epidemiologic role of rodent-borne *Bartonella* spp. as a cause of disease in humans is emerging in the Americas. This role has been suggested by identification of a novel rodent-associated *Bartonella* strain causing febrile illness in the rural southwestern United States (*10*) and a strain of pathogenic *B*. *elizabethae*, a bacteria that can cause human endocarditis, in the Huayllacallán Valley in Peru (*3*).

Because most identified *Bartonella* spp. have been reported as infectious agents for humans, our results should prompt public health concern. However, our findings require further investigation about the pathogenicity of these *Bartonella* genotypes. The detection of both pathogens in intradomestic and peridomestic areas where humans are in close contact with rodents could indicate that the incidence of both diseases in humans from Echarate District might be underestimated.
